# Adipogenic placenta-derived mesenchymal stem cells are not lineage restricted by withdrawing extrinsic factors: developing a novel visual angle in stem cell biology

**DOI:** 10.1038/cddis.2016.1

**Published:** 2016-03-17

**Authors:** C Hu, H Cao, X Pan, J Li, J He, Q Pan, J Xin, X Yu, J Li, Y Wang, D Zhu, L Li

**Affiliations:** 1 Collaborative Innovation Center for Diagnosis and Treatment of Infectious Diseases, State Key Laboratory for Diagnosis and Treatment of Infectious Diseases, School of Medicine, First Affiliated Hospital, Zhejiang University, Hangzhou, China

## Abstract

Current evidence implies that differentiated bone marrow mesenchymal stem cells (BMMSCs) can act as progenitor cells and transdifferentiate across lineage boundaries. However, whether this unrestricted lineage has specificities depending on the stem cell type is unknown. Placental-derived mesenchymal stem cells (PDMSCs), an easily accessible and less invasive source, are extremely useful materials in current stem cell therapies. No studies have comprehensively analyzed the transition in morphology, surface antigens, metabolism and multilineage potency of differentiated PDMSCs after their dedifferentiation. In this study, we showed that after withdrawing extrinsic factors, adipogenic PDMSCs reverted to a primitive cell population and retained stem cell characteristics. The mitochondrial network during differentiation and dedifferentiation may serve as a marker of absent or acquired pluripotency in various stem cell models. The new population proliferated faster than unmanipulated PDMSCs and could be differentiated into adipocytes, osteocytes and hepatocytes. The cell adhesion molecules (CAMs) signaling pathway and extracellular matrix (ECM) components modulate cell behavior and enable the cells to proliferate or differentiate during the differentiation, dedifferentiation and redifferentiation processes in our study. These observations indicate that the dedifferentiated PDMSCs are distinguishable from the original PDMSCs and may serve as a novel source in stem cell biology and cell-based therapeutic strategies. Furthermore, whether PDMSCs differentiated into other lineages can be dedifferentiated to a primitive cell population needs to be investigated.

Stem-cell-based therapies have gradually become a hot topic due to their high plasticity and self-renewing ability; clinical investigations with stem cell products in regenerative medicine are addressing a wide spectrum of conditions using a variety of stem cell types. These pluripotent cells including embryonic stem cells (ESCs), termed induced pluripotent stem cells (iPSCs), were first tested but inhibited in their clinical applications owing to ethical and tumorigenic problems. As a promising candidate for tissue regeneration, mesenchymal stem cells (MSCs) are fibroblast-like, with high plasticity and self-renewing ability and are able to develop into diverse cell lineages.^1^ Among the MSCs from different adult tissues, placental-derived mesenchymal stem cells (PDMSCs), which reside in the fetal membranes of the term placenta, are easily accessible and less invasive. Their abundance, high proliferative potency, short population doubling time, strong immunosuppression and lack of ethical concerns make them indispensable in stem cell research and therapy.^2^

Specific growth factors, cytokines and extracellular matrix components may have an important role in the determination of stem cell fate by switching from self-renewal to a differentiation stage. During lineage alteration to a specific tissue cell type, it was thought that MSCs progressively and developmentally became lineage restricted.^3^ Yet some evidences have suggested that when terminally differentiated mammalian cells are cultured under special conditions, they will revert to a more primitive phenotype.^4, 5, 6^ More recently, in the presence of human embryonic stem cell medium supplemented with valproic acid, stem cells derived from amniotic fluid could be fully reprogrammed to pluripotency without genetic manipulation.^7^ This process was defined as dedifferentiation and is considered as one of the mechanisms to reroute cell fate.^8^ Furthermore, a downregulation of lineage-specific genes and an upregulation of stem genes occurred immediately after initiation of the dedifferentiation process.^8^ This phase was characterized by repression of somatic genes via methylation, increased cell proliferation, altered morphology, signal transduction changes, reactivation of telomerase activity and the mesenchymal-to-epithelial transition (MET).^9, 10^ MET includes the loss of mesenchymal characteristics, such as motility, and the acquisition of epithelial characteristics such as cell polarity and the expression of cell adhesion molecules.^11^

In addition, bone marrow mesenchymal stem cells (BMMSCs) which were induced into osteocytes, chondrocytes and adipocytes, can dedifferentiate into a primitive population on the withdrawal of stimulating culture medium.^12, 13, 14^ This new population correlated with cell cycle arrest and associated genes, had enhanced cell survival, greater efficacy in differentiation and improved therapeutic potential *in vitro* and *in vivo* compared with uncommitted BMMSCs.^15, 16^ On the other hand, a number of studies showed enhanced mitochondrial biogenesis in various stem cell differentiation models including ESCs and iPSCs.^17, 18^ The immature mitochondrial phenotype in ESCs consists of fewer mitochondria, poorly developed cristae and a perinuclear location of mitochondria.^19, 20^ These characteristics have been regarded as potential markers of pluripotency in ESCs;^20^ however, it has not been clearly established whether the morphology and the mitochondrial network is pluripotency dependent or stem cell specific. In addition, it has been suggested that mitochondrial dynamics and oxidative phosphorylation (OXPHOS) activity can influence each other during the biological process.^21^ Consequently, we suggest that the altered OXPHOS activity will accompany the differentiation and dedifferentiation processes.

In the present study, we aimed to comprehensively analyze the transition in morphology, surface antigens, metabolism and multilineage potency during PDMSCs differentiation and dedifferentiation to clarify whether unrestricted lineage exists in differentiated PDMSCs. We showed that after withdrawing extrinsic factors, adipogenic PDMSCs reverted to a primitive cell population and retained stem cell characteristics. The new population proliferated faster than unmanipulated PDMSCs, and could be differentiated into adipocytes, osteocytes and hepatocytes. Gene expression profiling showed a panel of genes with significantly up- or downregulated expression between adipogenically differentiated and dedifferentiated cells. The cell adhesion molecules (CAMs) signaling pathway and extracellular matrix (ECM) components modulate cell behavior and enable the cells to regenerate, proliferate and differentiate during the differentiation, dedifferentiation and redifferentiation processes.^22, 23^ These observations indicated that the dedifferentiated PDMSCs were distinguishable from the original PDMSCs and may serve as a novel source in stem cell biology and cell-based therapeutic strategies.

## Results

### Morphology and adipocyte markers of adipogenic PDMSCs reverted to a primitive state after dedifferentiation

**Figure 1:**
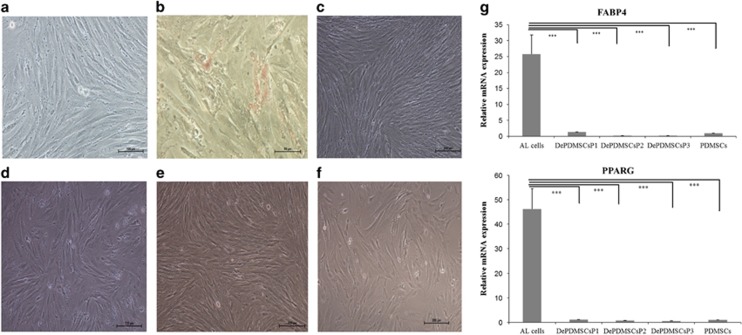
Morphology and adipocyte markers of adipogenic PDMSCs reverted to a primitive state after dedifferentiation. (**a**) Morphology of PDMSC P3 showed a long spindle shape. Scale bar, 100 *μ*m. (**b**) AL cells after Oil Red O staining demonstrated lipid accumulation. Scale bar, 50 *μ*m. (**c**) The nonstimulated controls were negative for Oil Red O staining. Scale bar, 200 *μ*m. (**d**) DePDMSCs in general PDMSCs medium at day 2. Scale bar, 100 *μ*m. (**e**) DePDMSCs in general PDMSCs medium at day 7. Scale bar, 100 *μ*m. (**f**) DePDMSCs at passage 3 occupied a similar shape as PDMSCs P3 in culture. Scale bar, 100 *μ*m. (**g**) RT-QPCR analysis of FABP4 and PPARG in PDMSCs, AL cells and DePDMSCs from passages 1 to 3. The expression of the PDMSC group was normalized to 1. Data are presented as the mean±S.D. in triplicate and were statistically analyzed by one-way ANOVA (*n*=3 independent donor cells); ***P<0.001

PDMSCs at passage 3 displayed a long spindle shape (Figure 1a). After adipogenesis for 14 days, PDMSCs exhibited the round shape characteristics of cultured adipocyte (Figure 1b) and thus were termed as adipocyte-like (AL) cells. AL cells showed an initiation of lipid droplet formation as shown by positive Oil Red O staining (Figure 1b). The nonstimulated controls were negative for Oil Red O staining (Figure 1c). However, withdrawal of adipogenic medium rapidly reverted AL cells to the long spindle morphology from day 2 to day 7 (Figures 1d and e). Then, the dedifferentiated PDMSCs was passaged (Figure 1f) and cultured in general PDMSC medium for another 14 days. At the molecular level, the expression of the adipocyte-specific genes FABP4 and PPARG were significantly upregulated at day 14 of adipogenesis when compared with undifferentiated PDMSCs (*P*<0.001). After withdrawing adipogenic medium, the markers were significantly downregulated to a similar level as PDMSCs, and there was no difference between each passage of dedifferentiated PDMSCs (Figure 1g).

### Surface antigen expression of adipogenic PDMSCs at the mRNA and protein levels reverted to a primitive state after dedifferentiation

**Figure 2:**
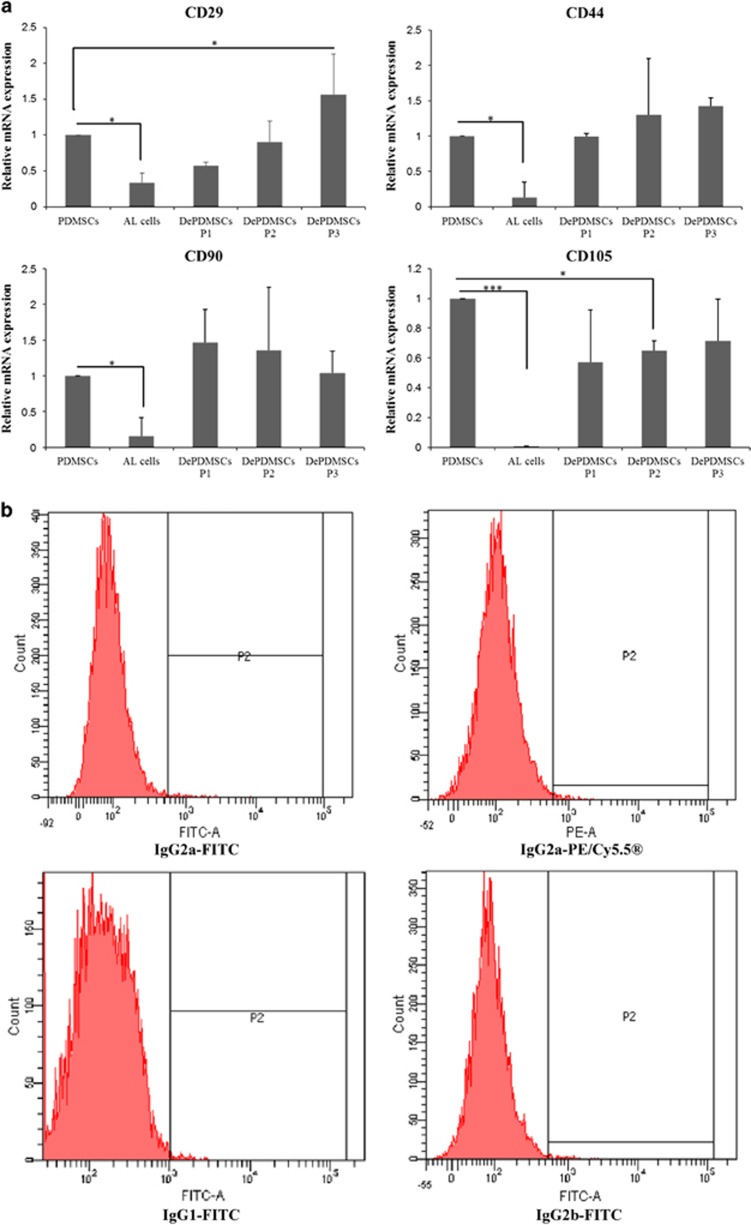

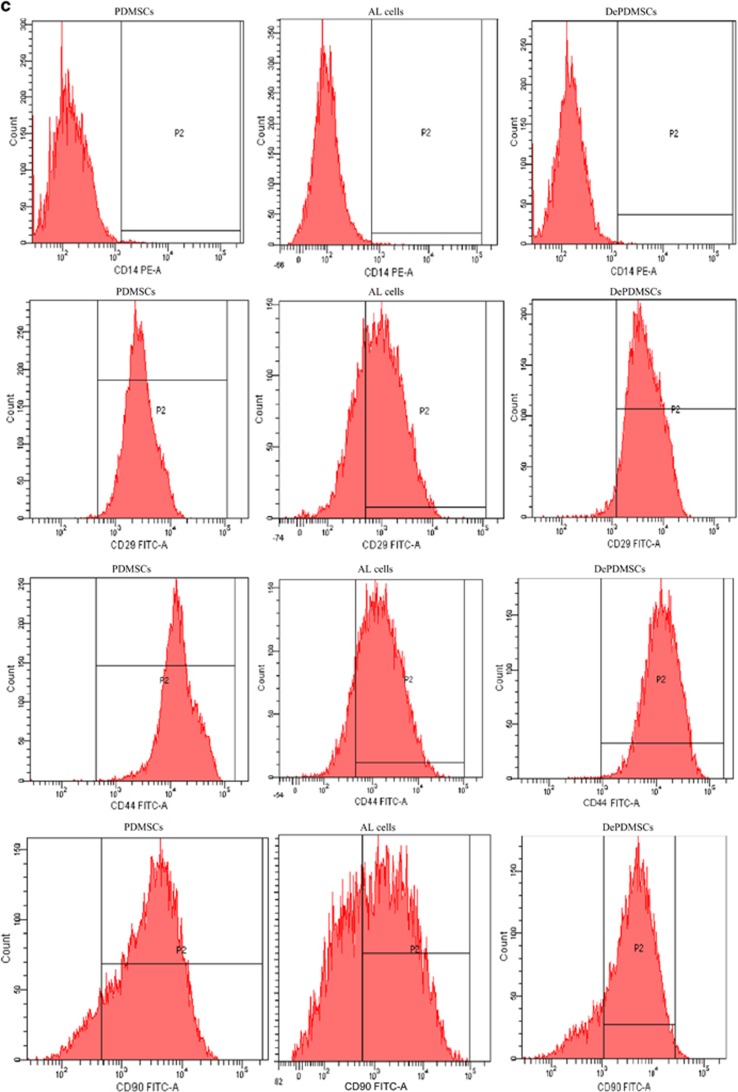

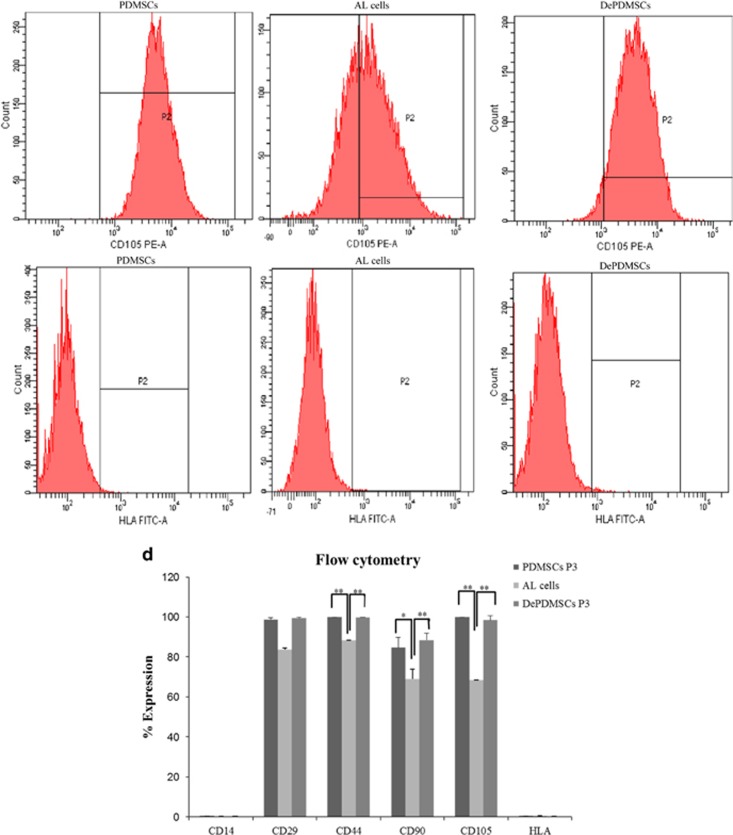

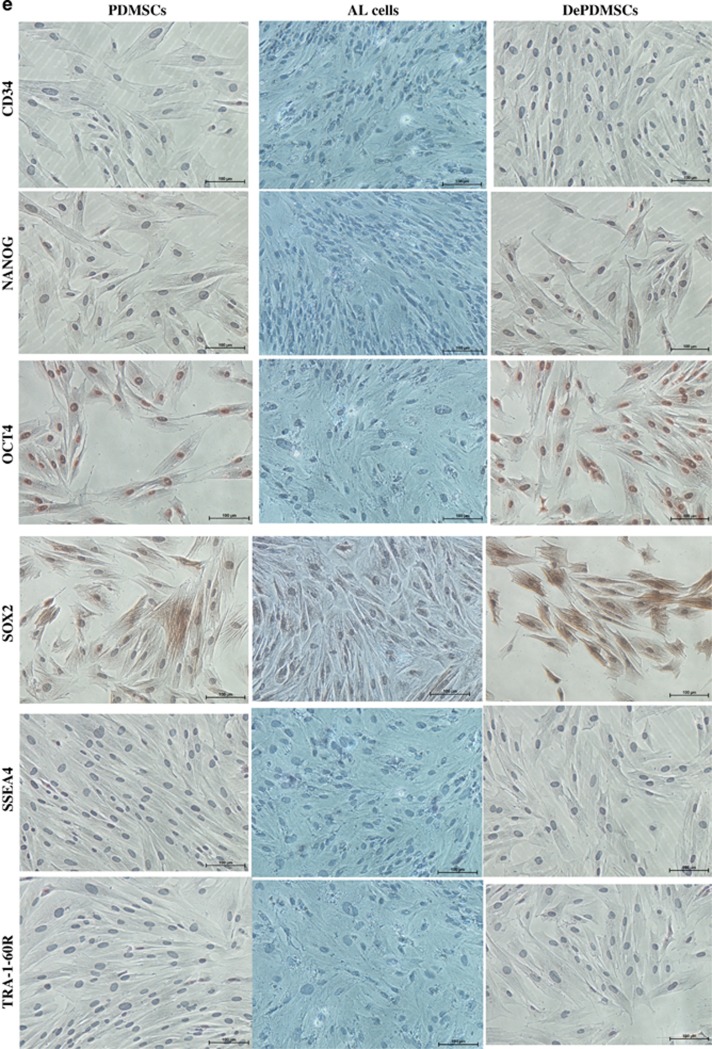
Positive surface antigens and ESC markers expression of DePDMSCs reverted to a primitive state, but the negative markers were not altered all the time. (**a**) RT-QPCR analysis of the expression of CD29, CD44, CD90 and CD105 on PDMSCs P3, AL cells and DePDMSCs from passages 1 to 3. (**b**) Immunophenotype of the negative controls determined by flow cytometry using labeled antibodies. (**c**) Immunophenotype of PDMSCs at passage 3, AL cells and DePDMSCs at passage 3 determined by flow cytometry using labeled antibodies specific for the indicated human surface antigens. (**d**) FACS analysis of PDMSCs at passage 3, AL cells and DePDMSCs at passage 3. Data are presented as the mean±S.D. in triplicate and were statistically analyzed by one-way ANOVA (*n*=3 independent donor cells); *P<0.05, **P<0.01. (**e**) CD34 and the embryonic stem cell markers expression of PDMSCs and DePDMSCs determined by immunocytochemistry; scale bars, 100 *μ*m

As previously reported, PDMSCs positively express mesenchymal markers such as CD29, CD44, CD90 and CD105, but negatively express the hematopoietic markers CD14 and HLA-DR.^24^ The expression levels in PDMSCs were set to 1 for normalization. Positive markers for CD29, CD44, CD90 and CD105 mRNA expression levels were significantly decreased in AL cells and then increased after dedifferentiation in general PDMSC medium (Figure 2a). The CD29 mRNA level in DePDMSCs at passage 3 was significantly higher than the level in PDMSCs at passage 3 (*P*<0.05). The CD105 mRNA level in AL cells was significantly downregulated compared with PDMSCs at passage 3 (*P*<0.001), and the mRNA expression level in DePDMSCs at passage 2 was lower than PDMSCs at passage 3 (*P*<0.05). However, the expression returned to a level comparable to PDMSCs at passage 3 after culture in general PDMSC medium for 21 days; cells at this stage were termed dedifferentiated PDMSCs (DePDMSCs) at passage 3 and were used for the subsequent analysis.

The immunophenotype of the negative controls was determined by flow cytometry using labeled antibodies (Figure 2b). The immunophenotype of PDMSCs, AL cells and DePDMSCs at passage 3 was determined by flow cytometry (Figure 2c). PDMSCs showed positive expression for CD29, CD44, CD90 and CD105, but negative expression for CD14 and HLA-DR. After adipogenic differentiation for 14 days, AL cells showed relatively lower expression for the positive markers in comparison with PDMSCs at passage 3; the negative makers were not altered. Then, after dedifferentiation for 21 days, DePDMSCs at passage 3 had a comparable expression pattern of positive and negative surface antigens to PDMSCs at passage 3. The results in our study showed that CD44, CD90 and CD105 were significantly altered during adipogenic differentiated PDMSCs (*P*<0.05) but reverted to a comparable level after dedifferentiation; the hematopoietic markers were not altered throughout the differentiation and dedifferentiation processes (Figure 2d).

Immunocytochemistry (*n*=3; Figure 2e) of the PDMSCs at passage 3 revealed highly positive expression for SOX2, weakly positive expression for OCT4 and negative expression for CD34, NANOG, SSEA4 and TRA-1-60R. After adipogenic differentiation for 14 days, AL cells showed downregulated expression for the positive embryonic stem cell markers (OCT4 and SOX2), while they returned to a level similar to uncommitted PDMSCs after dedifferentiation for 21 days. Conversely, the negative markers were not altered during the adipogenic differentiation and dedifferentiation processes.

### The mitochondrial network of PDMSCs undergoes changes during adipogenic differentiation and dedifferentiation

**Figure 3:**
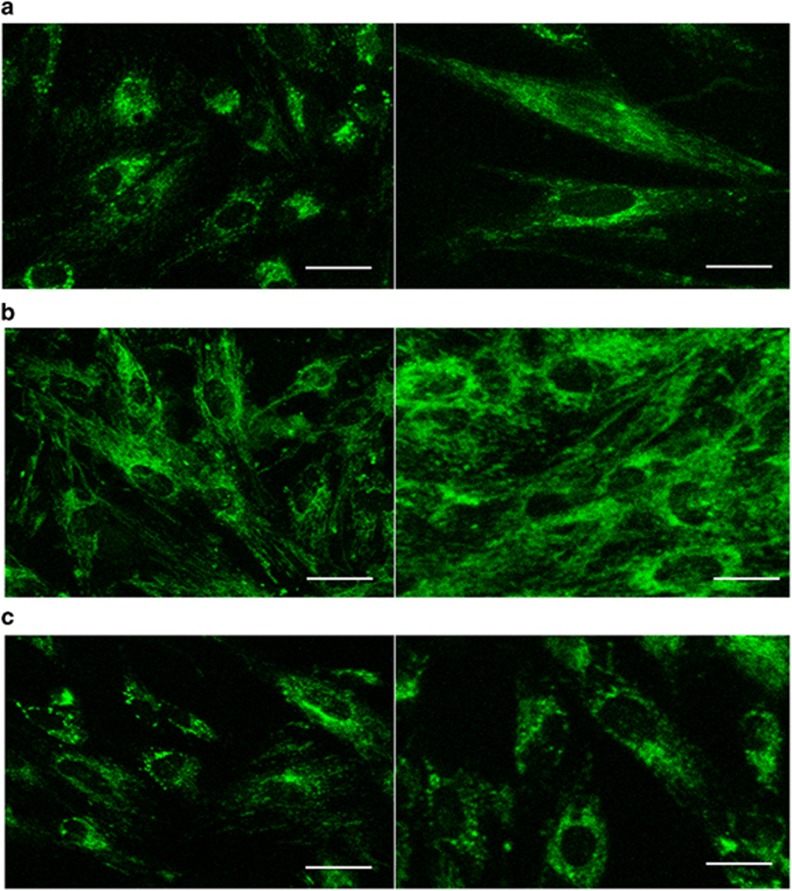

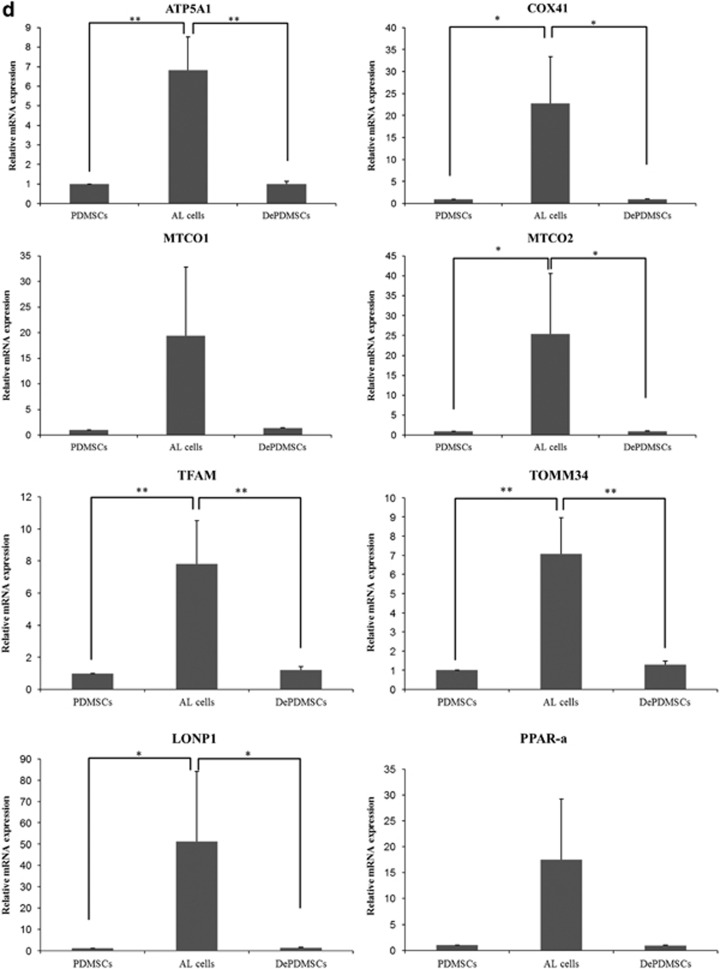

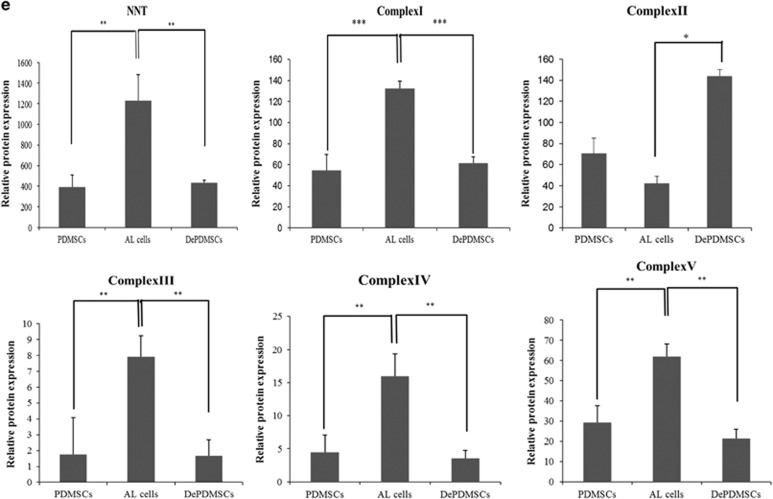
The mitochondrial network of PDMSCs underwent changes during adipogenic differentiation and dedifferentiation. Representative bright-field micrographs of (**a**) PDMSCs at passage 3, (**b**) AL cells and (**c**) DePDMSCs at passage 3 stained with MitoTracker Green FM (green); left, scale bars, 100 *μ*m; right, scale bars, 50 *μ*m. (**d**) RT-QPCR demonstrated increased mRNA expression of several mitochondrial biogenesis regulators after the adipogenic differentiation of PDMSCs, and these specific genes were downregulated after dedifferentiation. The expression levels of these genes in PDMSCs were normalized to 1. (**e**) OXPHOS related proteins expression of PDMSCs, AL cells and DePDMSCs were determined by the OXPHOS Magnetic Bead Panel. Data are presented as the mean±S.D. in triplicate and were statistically analyzed by one-way ANOVA (*n*=3 independent donor cells); *P<0.05, **P<0.01, ***P<0.001

Although the globular shape and perinuclear localization of mitochondria in ESCs and iPSCs has been regarded as a potential marker of pluripotency,^20^ it has not been clearly established whether the morphology of the mitochondrial network is pluripotency dependent or stem cell specific. Staining of the mitochondrial network revealed that expanding PDMSCs display a developed network composed of thread-like mitochondria spread throughout the cytoplasm (Figure 3a); adipogenic differentiation resulted in cells with more and larger round-shaped mitochondria (Figure 3b). After dedifferentiation, DePDMSCs also displayed a mitochondrial population similar to PDMSCs with a simultaneously decreased quantity (Figure 3c).

Given that mitochondrial biogenesis also requires the synthesis and import of many mitochondrial proteins, we next analyzed the abundance of several mitochondria specific genes at the mRNA level throughout the processes. These regulators included ATP5A1, COX4I1, MT-CO1, MT-CO2, TFAM, TOMM34, LONP1 and PPAR-a. We showed a trend towards mRNA abundance of all mitochondria specific genes in AL cells when compared with PDMSCs at passage 3, but they were downregulated in DePDMSCs at passage 3 (Figure 3d). ATP5A1, TFAM and TOMM34 in AL cells showed a significantly increased level compared with undifferentiated PDMSCs or DePDMSCs (*P*<0.01). COX4I1, MT-CO2 and LONP1 in AL cells also showed a significantly increased level compared with undifferentiated PDMSCs or DePDMSCs (*P*<0.05). However, MT-CO1 and PPAR-a demonstrated a higher trend but there was no difference in AL cells when compared with PDMSCs and DePDMSCs. These data further support the notion that an enhanced or decreased mitochondrial biogenesis process occurs during the differentiation and dedifferentiation processes.

OXPHOS is the main source of energy in eukaryotic cells. The results of a Human OXPHOS Magnetic Bead Panel (Figure 3e) showed a significantly increased trend for the nicotinamide nucleotide transhydrogenase (NNT) protein during the adipogenic process, but it decreased to the original level after dedifferentiation (*P*<0.01). Complex I was upregulated in AL cells when compared with PDMSCs but downregulated in DePDMSCs (*P*<0.001); Complex III, Complex IV and Complex V demonstrated similar alterations during the differentiation and dedifferentiation processes (*P*<0.01). Although the complex II protein level was lower in AL cells when compared with DePDMSCs at passage 3 (*P*<0.05), there was no difference between AL cells and PDMSCs. With the exception of NNT, the other OXPHOS proteins were generally at a low level during the differentiation and dedifferentiation processes.

### DePDMSCs can grow more quickly than uncommitted PDMSCs and expanding PDMSCs

**Figure 4:**
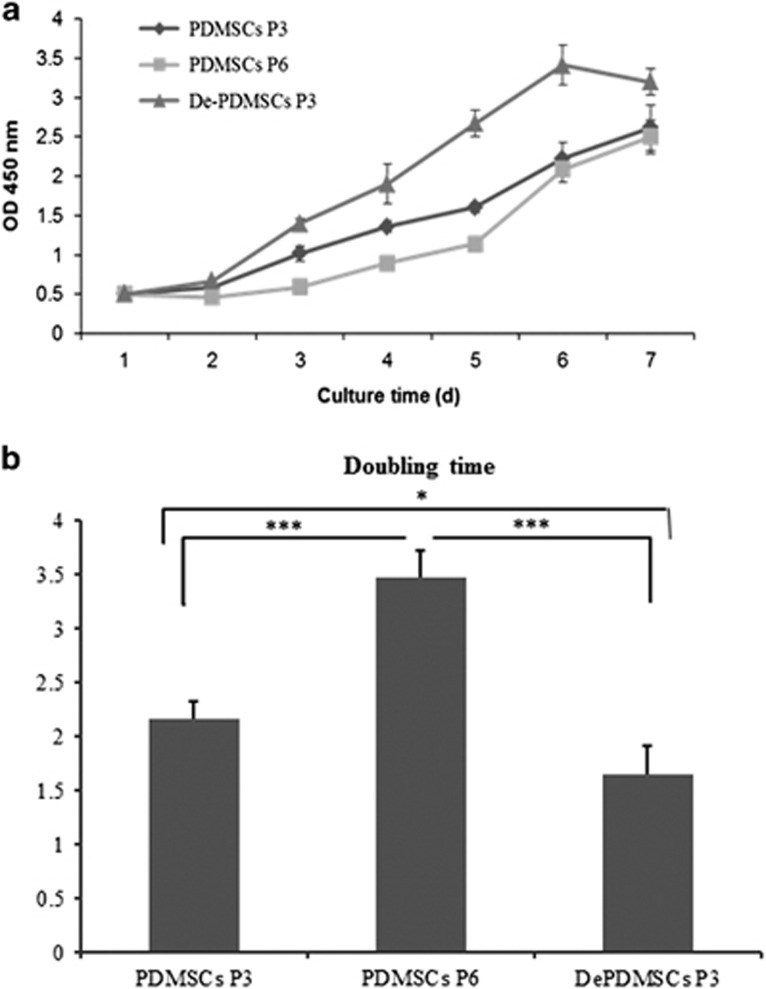
DePDMSCs can grow more quickly than unmanipulated PDMSCs and expanding PDMSCs. The growth curves of PDMSCs P3, PDMSCs P6 and DePDMSCs P3 were detected by a Cell Proliferation Reagent WST-1 assay at 450 nm on a spectrophotometer (*n*=3). (**a**) The proliferation rates of PDMSCs at passage 3, PDMSCs at passage 6 and DePDMSCs at passage 3. (**b**) The doubling time of PDMSCs at passage 3, PDMSCs at passage 6 and DePDMSCs at passage 3. Data are presented as the mean±S.D. in triplicate and were statistically analyzed by one-way ANOVA (*n*=3 independent donor cells); *P<0.05, ***P<0.001

We further detected the proliferation ability of DePDMSCs and compared this with uncommitted PDMSCs and expanding PDMSCs. We found that the proliferation rates (Figure 4a) of PDMSCs at passage 3, PDMSCs at passage 6 and DePDMSCs at passage 3 were slow during the first 2–3 days (latent phase) and then accelerated rapidly during 4–6 days (logarithmic phase) and thereafter slowed down (stationary phase). The doubling time of PDMSCs at passage 3 in the logarithmic phase was 2.16±0.16d, PDMSCs at passage 6 was 3.46±0.25d and DePDMSCs at passage 3 was 1.65±0.26d (Figure 4b). The proliferation ability results showed that DePDMSCs at passage 3 grew more quickly than PDMSCs at passage 3 (*P*<0.05). The proliferation ability of DePDMSCs at passage 3 and PDMSCs at passage 3 was higher than PDMSCs at passage 6 (*P*<0.001).

### The multilineage differentiation ability of DePDMSCs was comparable to PDMSCs

To clarify whether the new dedifferentiated population was able to obtain multilineage differentiation ability, we further induced the new population via adipogenic, osteogenic and hepatogenic differentiation and compared them with PDMSCs.

**Figure 5:**
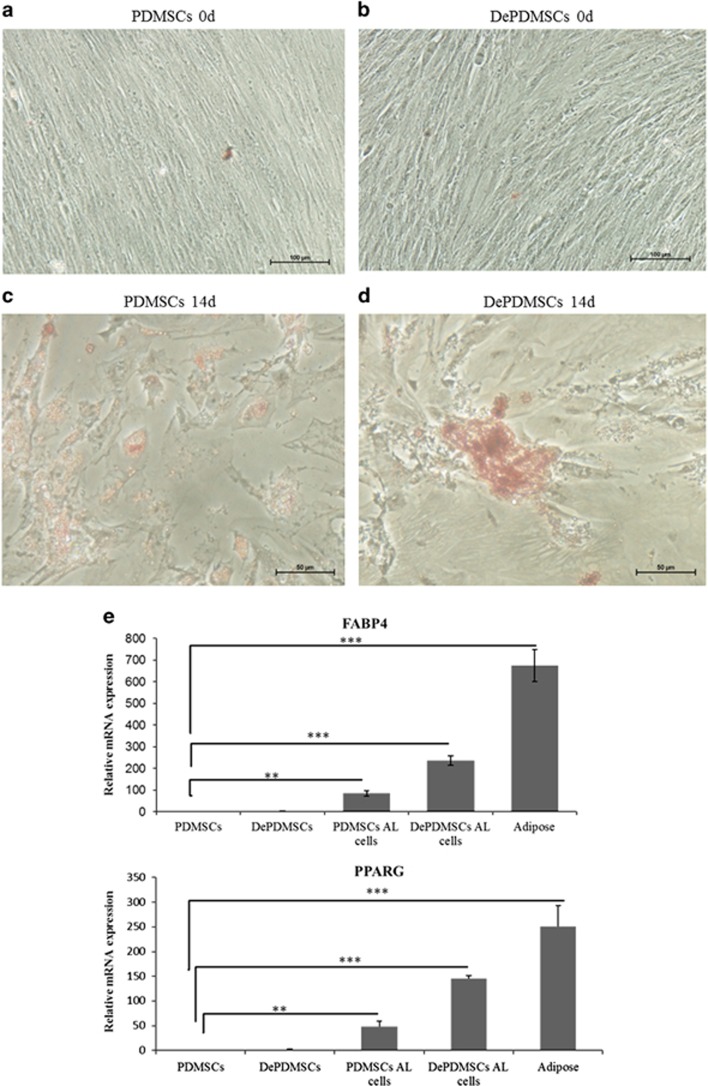

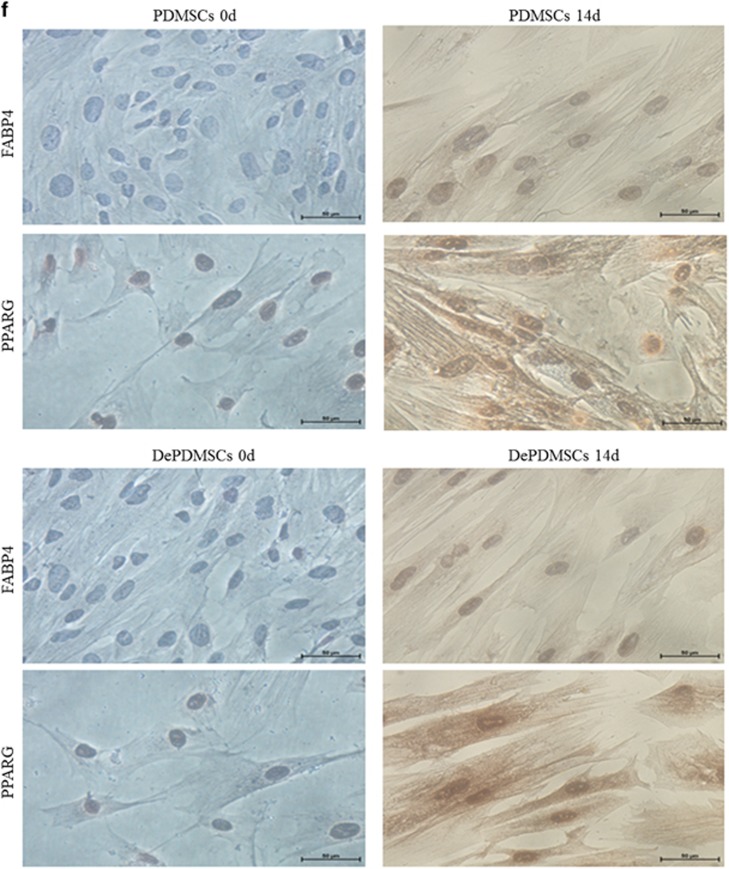
Adipogenic differentiation ability in DePDMSCs showed a greater trend than PDMSCs. (**a**) Oil red O staining of PDMSCs maintained in normal growth medium; scale bar, 100 *μ*m. (**b**) Oil red O staining of DePDMSCs maintained in normal growth medium; scale bar, 100 *μ*m. (**c**) Oil red O staining of PDMSCs that had undergone adipogenic differentiation at 14d (lipid accumulation); scale bar, 50 *μ*m. (**d**) Oil red O staining of DePDMSCs that had undergone adipogenic differentiation at 14d (lipid accumulation); scale bar, 50 *μ*m. (**e**) Representative RT-QPCR analyses of PDMSCs and DePDMSCs cultured in adipogenic medium for 14 days. Human adipose tissue was used as a positive control. The expression levels of FABP4 and PPARG in PDMSCs were normalized to 1. (**f**) Immunocytochemistry of FABP4 and PPARG showed negative expression in PDMSCs and DePDMSCs on day 0 and strongly positive expression on day 14; scale bar, 50 *μ*m. Data are presented as the mean±S.D. in triplicate and were statistically analyzed by one-way ANOVA (*n*=3 independent donor cells); **P<0.01, ***P<0.001

After PDMSCs and DePDMSCs were induced in adipogenic medium for 14 days, they acquired the typical characteristics of adipocytes, and Oil red O staining showed lipid accumulation (Figures 5a and d). DePDMSCs retained adipogenic potency and the mRNA expression levels of FABP4 and PPARG in adipogenic DePDMSCs were higher than adipogenic PDMSCs. Human adipose tissue was used as a positive control (Figure 5e). Immunocytochemistry of FABP4 and PPARG showed negative expression in PDMSCs and DePDMSCs at day 0, and strongly positive expression at day 14 of adipogenesis (Figure 5f).

**Figure 6:**
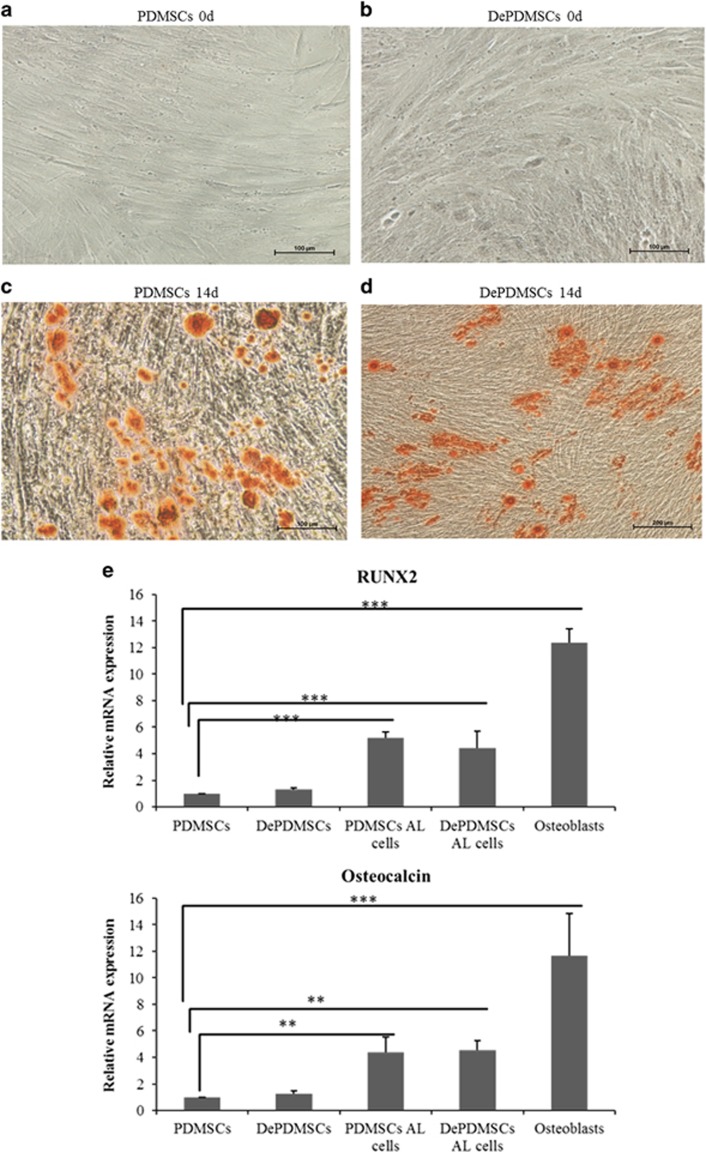

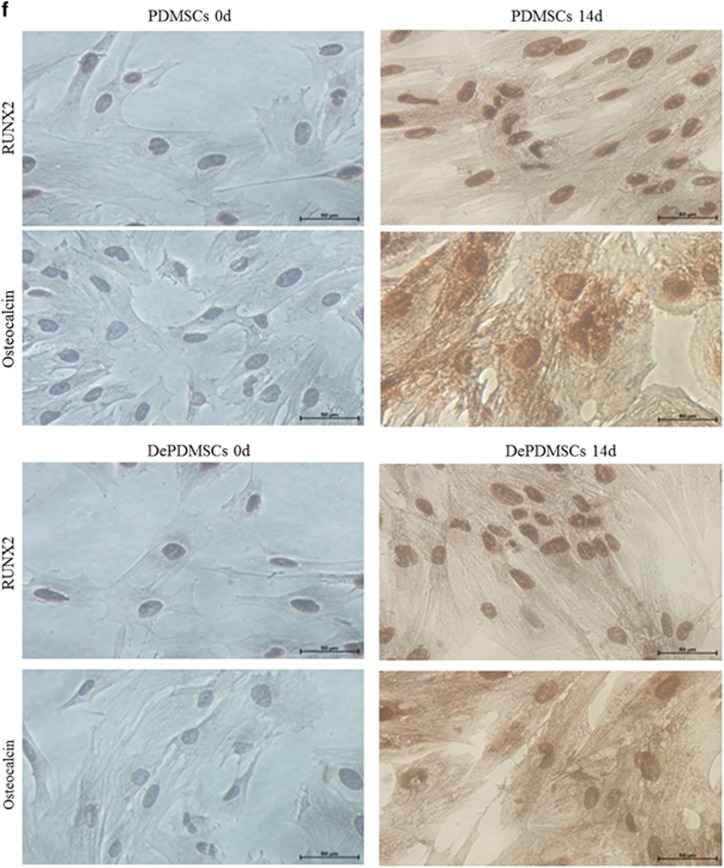
The osteogenic differentiation ability in DePDMSCs was comparable to PDMSCs. (**a**) Alizarin red staining of PDMSCs maintained in normal growth medium; scale bar, 100 *μ*m. (**b**) Alizarin red staining of DePDMSCs maintained in normal growth medium; scale bar, 100 *μ*m. (**c**) Alizarin red staining of PDMSCs that had undergone osteogenic differentiation at 14d (bone nodules formed); scale bar, 100 *μ*m. (**d**) Alizarin red staining of DePDMSCs that had undergone osteogenic differentiation at 14d (bone nodules formed); scale bar, 200 *μ*m. (**e**) Representative RT-QPCR analyses of PDMSCs and DePMSCs cultured in osteogenic medium for 14 days. Osteoblasts were used as a positive control. The expression levels of RUNX2 and OC in PDMSCs were normalized to 1. (**f**) Immunocytochemistry of RUNX2 and OC showed negative expression in PDMSCs and DePDMSCs on day 0 and strongly positive expression on day 14; scale bar, 50 *μ*m. Data are presented as the mean±S.D. in triplicate and were statistically analyzed by one-way ANOVA (*n*=3 independent donor cells); **P<0.01, ***P<0.001

After PDMSCs and DePDMSCs were induced in osteogenic medium for 14 days, they acquired the typical characteristics of osteocytes, and alizarin red staining showed calcium accumulation (Figures 6a and d). DePDMSCs retained osteogenic potency and the mRNA expression levels of RUNX2 and osteocalcin in osteogenic DePDMSCs were comparable to osteogenic PDMSCs. Osteoblasts were used as a positive control (Figure 6e). Immunocytochemistry of RUNX2 and osteocalcin showed negative expression in PDMSCs and DePDMSCs at day 0, and strongly positive expression at day 14 of osteogenesis (Figure 6f).

**Figure 7:**
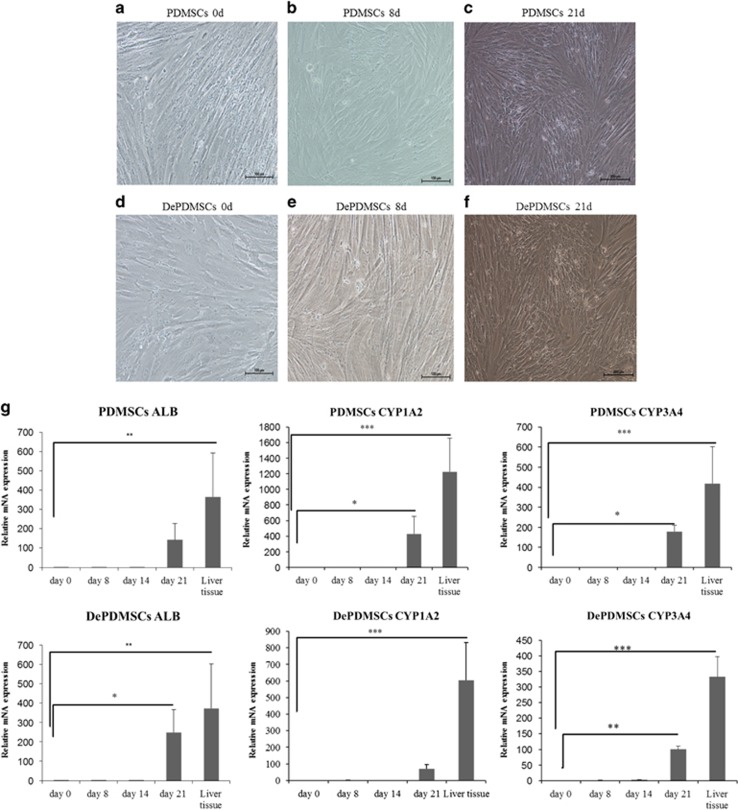

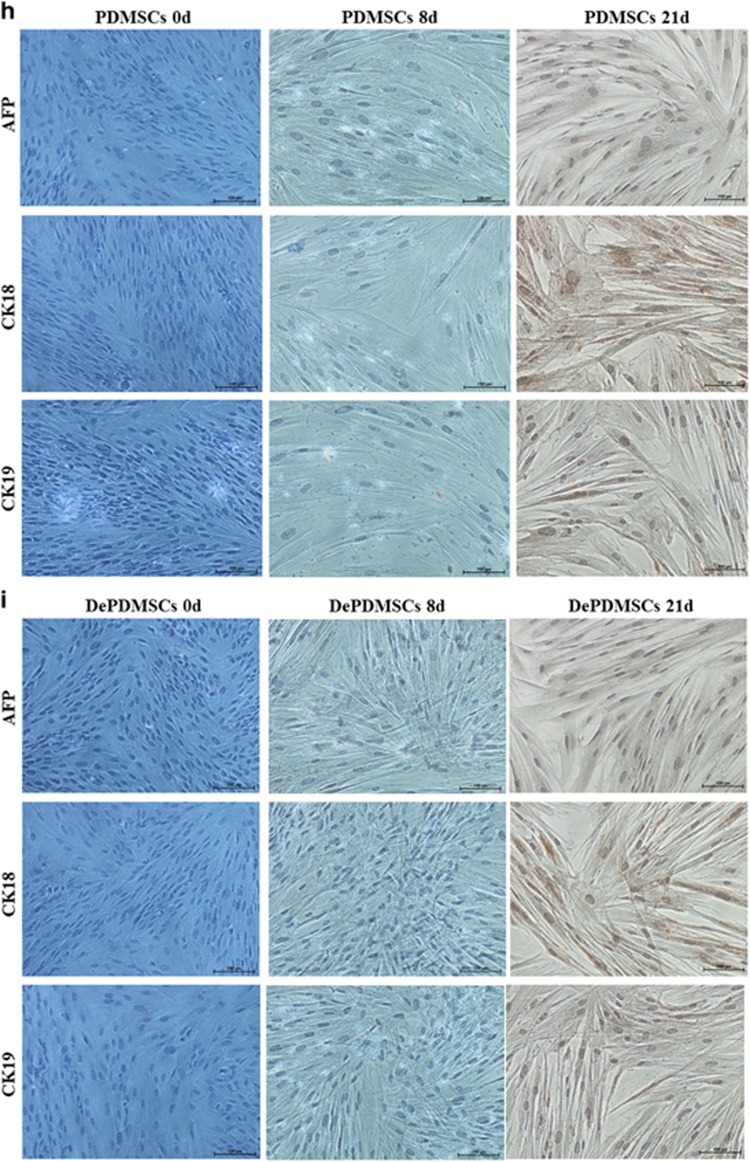

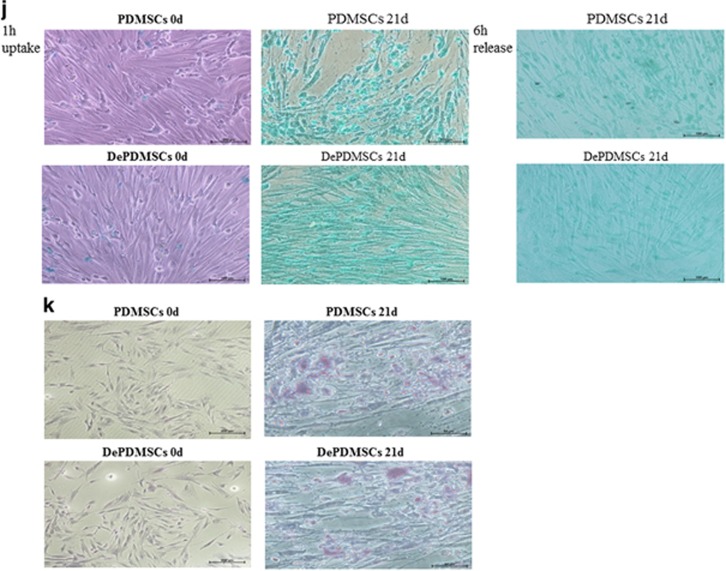
The hepatogenic differentiation ability in DePDMSCs was comparable to PDMSCs. (**a**) Hepatocyte differentiation of PDMSCs at day 0; scale bar, 100 *μ*m. (**b**) Hepatocyte differentiation of PDMSCs at day 8; scale bar, 100 *μ*m. (**c**) Hepatocyte differentiation of PDMSCs at day 21; scale bar, 200 *μ*m. (**d**) Hepatocyte differentiation of DePDMSCs at day 0; scale bar, 100 *μ*m. (**e**) Hepatocyte differentiation of DePDMSCs at day 8; scale bar, 100 *μ*m. (**f**) Hepatocyte differentiation of DePDMSCs at day 21; scale bar, 200 *μ*m. (**g**) RT-QPCR analysis of ALB, CYP1A2 and CYP3A4 in hepatogenic PDMSCs and DePDMSCs on days 0, 8, 14 and 21. Liver tissue was as a positive control. The undifferentiated PDMSC group or undifferentiated DePDMSCs was normalized to 1. The mRNA expression levels of CYP1A2 and CYP3A4 in PDMSCs were significantly increased after hepatogenic induction for 21d (*P*<0.05). The mRNA expression levels of ALB (*P*<0.05) and CYP3A4 (*P*<0.01) in DePDMSCs were significantly increased after hepatogenic induction for 21d. Data are presented as the mean±S.D. in triplicate and were statistically analyzed by one-way ANOVA (*n*=3 independent donor cells); *P<0.05, **P<0.01, ***P<0.001. (**h**) Immunocytochemistry of AFP, CK18 and CK19 showed negative expression in PDMSCs at day 0, weakly positive expression at day 8, and strongly positive expression at day 21. (**i**) Immunocytochemistry of AFP, CK18 and CK19 showed negative expression in DePDMSCs at day 0, weakly positive expression at day 8 and strongly positive expression at day 21; scale bars, 100 *μ*m. (**j**) Uptake of ICG on hepatogenic PDMSCs and DePDMSCs at day 0 and day 21 and secretion of ICG from hepatogenic PDMSCs and DePDMSCs at day 21; scale bars, 100 *μ*m. (**k**) Periodic acid staining for glycogen on hepatogenic PDMSCs and DePDMSCs at day 0 (scale bar, 200 *μ*m) and day 21 (scale bar, 50 *μ*m)

After PDMSCs and DePDMSCs were induced stepwise in hepatogenic medium for 21 days, the long spindle cells gradually turned to the classical cubic morphology of hepatocytes (Figures 7a and f). RT-QPCR analysis revealed significantly upregulated mRNA expression of ALB, CYP1A2 and CYP3A4 in PDMSCs and DePDMSCs at day 21 of hepatogenic differentiation. Human liver tissue was used as a positive control (Figure 7g). Immunocytochemistry (Figures 7h and i) revealed the presence of hepatic markers (AFP, CK18 and CK19) in hepatogenic PDMSCs and DePDMSCs that was weakly positive at day 8 and of moderate intensity at day 21. Hepatogenic PDMSCs and DePDMSCs could also uptake and release ICG; however, the expanding PDMSCs and DePDMSCs could not uptake ICG (Figure 7j). Periodic acid-Schiff staining (PAS) staining showed that both PDMSCs and DePDMSCs could store more glycogen after hepatogenic induction for 21d (Figure 7k).

### Numerous genes and multiple signaling pathways cooperate to regulate the adipogenic differentiation and dedifferentiation processes

Gene expression profiling was performed to obtain a deeper molecular insight into AL cells and their subsequent dedifferentiation to primitive cell types with multilineage potency. GeneChips were generated for PDMSCs, AL cells and DePDMSCs from three donors. We selected 2140 out of 49 395 probe sets that represented genes with differential expression between AL cells and PDMSCs after removing double entries and probe sets with no title (Supplementary Table S1). Among them, the expression levels of 952 genes were upregulated and 1 188 were downregulated on day 14 of adipogenesis. Using the same criteria, we selected 2486 out of 49 395 probe sets that represented genes with differential expression between AL cells and DePDMSCs (Supplementary Table S2). Among them, the expression levels of 1590 genes were upregulated and 896 were downregulated on day 21 of dedifferentiation. On the basis of KEGG pathway enrichment analysis, the upregulated and downregulated differentially expressed genes were mainly enriched in the multiple crucial KEGG pathways listed in Tables 1a and b. We then identified nine differentially expressed genes in DePDMSCs compared with PDMSCs (Supplementary Table S3), all genes except for fibroblast growth factor 7 (FGF7) were upregulated in DePDMSCs when compared with PDMSCs. KEGG pathway enrichment analysis demonstrated that three of the differentially expressed genes were involved in multiple pathways (Table 1c). To confirm the microarray analysis results, RT-QPCR analysis (Supplementary Figure S1) showed that the expression levels of most genes in DePDMSCs were consistent with the microarray data except for FGF7. Herein, the expression of cell adhesion molecule 1 (CADM1) was upregulated in DePDMSCs more than sevenfold when compared with PDMSCs (*P*<0.001). Meanwhile, the expression of matrix metallopeptidase 10 (MMP10) in DePDMSCs was upregulated for more than sevenfold (*P*<0.01), the expression of zinc finger protein 711 (ZNF711) in DePDMSCs was upregulated for more than threefold (*P*<0.05). Among them, CADM1 was included in the CAMs signaling pathway and could be beneficial to identify the central nodes within the signaling web of ECM on functional basis.^25^

## Discussion

In contrast to the lineage restriction in differentiated cells, it has been reported that cell fate is interconvertible.^26, 27^ Just through ceiling culture and without the addition of cytokines, isolated adipocytes can undergo dedifferentiation and acquire multilineage differentiation potency.^28, 29^ However, it is still unclear whether this phenomenon is a hallmark of cell differentiation programs or displays specificities depending on the stem cell types.

Despite being morphologically and phenotypically similar to uncommitted PDMSCs, DePDMSCs represent a previously undescribed distinct population of stem cells with several distinguished features. A previous study showed that the expression levels of ESCs markers were gradually upregulated after dedifferentiation of adipogenic amniotic fluid stem cells.^30^ Generally speaking, stem cells became static and had nearly no proliferative activity after differentiation. It is generally accepted that decreased cell-division ability accompanies long culture times and increased age.^31^ Considering the intriguing results in our study, AL cells may return to the cell cycle and proliferate quickly after dedifferentiation, indicating that this proliferative restriction is not permanent and dedifferentiation activates cell cycle progression genes for subsequent proliferation and transdifferentiation.^15^

Recent studies performed on ESCs and iPSCs showed enhanced mitochondrial biogenesis after differentiation,^18^ but whether the mitochondrial biogenesis in various stem cell models is similar to ESCs or iPSCs is not clear. Mitochondria are cytoplasmic organelles that have a primary role in cellular metabolism and homeostasis, the regulation of the cell signaling and programmed cell death.^32^ Some reports discovered a similar phenomenon in MSCs, but this was limited to BMMSCs.^33, 34, 35, 36, 37, 38^ Studies of adipogenic or osteogenic differentiation of BMMSCs also showed an increase in mitochondrial biogenesis and function (an increased mRNA abundance for MT-CO1, MT-CO2, COX4I1 and ATP5A1) during early steps of the differentiation process.^36, 39^ Furthermore, evidence also indicates that mitochondrial biogenesis is strongly associated with differentiation, and is accompanied with increased expression of subunits from complexes I, II and III, and a higher mitochondrial activity by significantly increased oxygen consumption.^40^ As a consequence, differentiated cells displayed increased mitochondrial mass, a more developed mitochondrial network, and a shift toward OXPHOS to meet their energy demands.^41, 42, 43^ Besides, NNT functions as a high-capacity source of mitochondrial nicotinamide adenine dinucleotide phosphate (NADPH), the mutation results in mitochondrial redox abnormalities, most notably a poor ability to sustain NADP and glutathione in their reduced states, ultimately resulting in increased cellular oxidative stress and impaired morphology and mitochondrial function.^44, 45^ These co-regulators cooperate to drive mitochondrial biogenesis and oxidative switching by co-activating many transcription factors. In fact, the use of molecules to promote or inhibit mitochondrial biogenesis or function, or by interfering with the expression of mitochondrial biogenesis regulators or proteins involved in mitochondrial function, has been demonstrated to impact stemness and cell differentiation.^18, 46, 47^

To determine whether the converted cells with similar phenotypes are just structural entities or retain their multilineage potency requires further investigation. Then, we converted DePDMSCs to adipocytes, osteocytes and hepatocytes. A previous study showed that the dedifferentiated adipocytes had adipogenic potency,^29^ the results of our study suggest greater adipogenic potency in DePDMSCs than in PDMSCs. Furthermore, another study demonstrated that the dedifferentiated cells could achieve the morphology of other lineages more easily and quickly during the process of transdifferentiation.^48^ On the other hand, dedifferentiated BMMSCs can redifferentiate into neural cells, osteocytes and adipocytes,^15, 16^ the dedifferentiated PDMSCs in our study were able to overcome the mesodermal commitment to other lineages. However, there is no study on the detection of hepatocyte differentiation potency in dedifferentiated MSCs as far as we know. The hepatogenic differentiation ability of DePDMSCs is comparable to PDMSCs in our study. In sum, the successful fate conversion of dedifferentiated PDMSCs was not restricted to related lineages within the same germ layer but notably crossed the lineage boundaries beyond limited cellular conversion.^49^

The sequential adipogenic differentiation and dedifferentiation processes resulted in novel stem cells that proliferated faster and retained multilineage potency; however, the mechanism underlying this cross-talk remained to be determined. In this context, we identified a number of differentially expressed genes that were regulated by the complex communication between signaling pathways during adipogenic differentiation and dedifferentiation. Intriguingly, we found a single gene named CADM1 was extremely higher in DePDMSCs when compared with PDMSCs. CADM1 can directly regulate mast cell net adhesion directly through CADM1-dependent adhesion,^25^ furthermore, it was strongly correlated with the bone-forming capacity of human MSCs and could be used as a reliable *in vitro* diagnostic marker.^50^ CAMs serve as a well-known signaling pathway for diverse biological processes including cellular interactions, adhesions and micro-environmental decisions.^22^ In addition to that, ECM has a critical role in the formation of adipogenically differentiated cells and the differentiated cells started to release the lipid droplets and leave bare network of ECM.^51, 52^ The ECM components modulate cell behavior and enable the cells to regenerate, proliferate, differentiate, grow, orientate and constrain themselves for perfectible regeneration by cell–cell and cell–ECM interactions.^23^ Here, we indicated that the enhanced proliferative ability and differentiation ability of DePDMSCs may attribute to the upregulated CAMs signaling pathway and ECM components. In spite of that, further research is still required to unravel the process in other cell types and to clarify more detailed mechanisms involved in the interplay between these two processes, which progressed as a reprogramming method in ESCs or iPSCs.

In conclusion, we systematically and comprehensively demonstrated that PDMSC-derived AL cells were able to successfully dedifferentiate and acquire a more primitive phenotype under certain culture conditions. In addition, we not only detected alterations in morphology, adipocyte markers and stem cell markers, but we also observed the mitochondrial network during differentiation and dedifferentiation and found that it may serve as a marker of absent or acquired pluripotency in various stem cell models. Furthermore, the dedifferentiated cells entered the cell cycle and retained their multi-potentiality to transdifferentiate into other lineages in response to extrinsic factors. The CAMs signaling pathway and ECM components regulate cell behavior in proliferation and differentiation; consequently, we suggest that biochemical analysis of native adipogenic ECM would be a crucial guide for artificial designing of biomaterials. With respect to the clinical use of MSCs in regenerative medicine, the sequence differentiation, dedifferentiation, redifferentiation and transdifferentiation could be a vital approach to treat damaged or diseased tissues or to generate tissue-specific mature cells *in vitro* in the future.

## Materials and Methods

### Isolation and culturing of PDMSCs

Placentas were obtained from donors at the Hangzhou Red Cross Hospital in China after informed consent had been obtained. The study used a protocol approved by the Research Ethics Committee of the First Affiliated Hospital, School of Medicine, Zhejiang University. The placental tissue was washed with preheated phosphate-buffered saline (PBS, pH 7.2±0.1, GenomSciences, Hangzhou, China), minced and digested using 0.1% (w/v) collagenase type IV (Invitrogen Life Technologies, Carlsbad, CA, USA) at 37 °C for 30 min. Recovered cells and digested cell debris were filtered through a 100-*μ*m cell strainer. Mononuclear cells obtained by lymphocyte isolation (GE Healthcare, Ficoll-Pague PLUS, Uppsala, Sweden) were cultured in special medium (MesenCult Human Basal Medium plus MesenCult Human Supplement, STEMCELL Technologies, Vancouver, BC, Canada) and adjusted to 2 × 10^6^ cells in T25-cm2 tissue culture flasks (Nunc Flasks Nunclon ▵ with Filter Cap, Nunclon ▵ Surface, Roskilde, Denmark) maintained in an incubator at 37 °C in a humidified atmosphere with 5% (v/v) CO_2_. Approximately 4 to 6 days later, many colonies had formed. After achieving 60–70% confluence, adherent cells were trypsinized with 0.25% (w/v) trypsin/EDTA (Invitrogen Life Technologies) and re-plated at a 1:3 dilution. Placenta-derived cells were cultured continuously under the same conditions.

### Adipogenic differentiation and dedifferentiation of PDMSCs

To induce adipogenesis, the third passage PDMSCs at 1 × 10^5^/well density in 12-well plates were treated with adipogenic medium (OriCell hMSC Adipogenic Differentiation Medium, Cyagen Biosciences, Guangzhou, China) in 12-well plates for 2 weeks. The differentiated PDMSCs were defined as AL cells and then the adipogenic medium was removed from AL cells and replaced by general PDMSCs medium for 1 week. After that, the cells were defined as DePDMSCs at passage 1. Then, DePDMSCs continued to be cultured in general PDMSCs medium to passage 3. After 2 weeks, lipoprotein lipase (FABP4) and peroxisome proliferator-activated receptor-g (PPARG) were identified by mRNA detection.

### RNA extraction and reverse transcription

Using the RNAiso plus kit (TaKaRa, Tokyo, Japan), total RNA was isolated according to the manufacturer's instructions. The RNA was first treated with DNase (TaKaRa) in a 10 *μ*l reaction with 5 × gDNA Easer Buffer (2 *μ*l), gDNA Easer (1 *μ*l) and total RNA (1 *μ*g). The reaction was conducted at 42 °C for 2 min. For the mRNAs, the PrimeScript RT reagent Kit (TaKaRa) was used for reverse transcription (RT) in a total volume of 20 *μ*l with 4 *μ*l 5 × PrimeScript Buffer PCR buffer, 1 *μ*l PrimeScript RT enzyme mix I, 1 *μ*L RT Primer Mix and 10 *μ*l of the RNA sample. The RT reaction started with a 15-min incubation period at 37 °C and ended after a 5-s enzyme-denaturing step at 85 °C.

### Real-time quantitative-PCR

Real-time amplification was performed using the SYBR Premix Ex Taq (TaKaRa) in an ABI 7900 thermocycler (Applied Biosystems, Foster City, CA, USA). Real-time quantitative-PCR (RT-QPCR) was carried out in a 10 *μ*l reaction system containing 5 *μ*l SYBR Premix Ex Taq (2 ×), 0.4 *μ*l PCR primers (10 *μ*M), 0.2 *μ*l ROX reference dye and 1 *μ*l cDNA. PCR thermal cycling parameters were 95 °C for 30 s, followed by 40 cycles of 95 °C for 5 s and then 60 °C for 30 s. After PCR, a dissociation curve was constructed at 95 °C for 15 s, 60 °C for 15 s and 95 °C for 15 s for detection of PCR product specificity. Each reaction was performed in triplicate. Positive mesenchymal surface antigens and mitochondria, adipocyte, osteocyte and hepatocyte-specific genes were performed by RT-QPCR. The mitochondrial specific genes detected included ATP synthase, H+ transporters, mitochondrial F1 complex, alpha subunit 1 (ATP5A1), cytochrome *c* oxidase subunit IV isoform 1 (COX4I1), mitochondrially encoded cytochrome *c* oxidase I (MT-CO1), mitochondrially encoded cytochrome *c* oxidase II (MT-CO2), transcription factor A, mitochondrial (TFAM), translocase of outer mitochondrial membrane 34 (TOMM34), lon peptidase 1, mitochondrial (LONP1) and peroxisome proliferator-activated receptor alpha (PPAR-a). The adipocyte-specific genes including FABP4 and PPARG were detected. The osteocyte-specific genes including runt-related transcription factor 2 (RUNX2) and osteocalcin (OC) were detected. The hepatocyte-specific genes including cytochrome P (CYP)1A2, CYP3A4 and albumin (ALB) were detected. The differentially expressed genes of microarray in DePDMSCs and PDMSCs were confirmed by RT-QPCR. The reference gene *β*-actin was used as a relative control for the expression levels. The primers for the target products were designed as in Supplementary Table S4.

### Flow cytometry for surface antigen expression

Culture-expanded cells were washed with PBS-containing 0.3% (w/v) bovine serum albumin (BSA) and the concentration was adjusted to 1 × 10^6^ cells/100 *μ*l. PDMSCs at passage 3, AL cells and DePDMSCs at passage 3 were examined for mesenchymal and hematopoietic marker expression of surface antigens by incubating with the antibodies CD14-phycoerythrin (PE), CD29-fluorescein isothiocyanate (FITC), CD44-FITC, CD105-PE and HLA-DR-FITC (Abcam, Cambridge, UK). Antibodies including mouse IgG2a-FITC, mouse IgG2a-PE/Cy5.5, mouse IgG1-FITC and rat IgG2b-FITC (Abcam) were used as isotype controls. After being labeled with antibodies in the dark at room temperature for 30 min, cells were washed twice with PBS. Flow cytometry was conducted using a BD LSR II (Beckman Coulter, Los Angeles, CA, USA), and the data were analyzed using BD FACSDiva software.

### Mitochondria fluorescent measurement

Mito-Tracker Green FM (Invitrogen Life Technologies) was diluted in L-DMEM (Gibco, Carlsbad, CA, USA) to the appropriate concentrations. Cells were incubated with pre-warmed (37 °C) probe containing medium for 60 min for staining. PDMSCs at passage 3, AL cells and DePDMSCs at passage 3 were visualized and photographed on a fluorescent microscope (ZEISS LSM710, Jena BioSciences, Jena, Germany). Five different areas from each image were taken.

### Cell lysis collection

Cells were rinsed with ice-cold PBS, and then ice-cold mitochondrial lysis buffer with freshly added phosphatase and protease inhibitors were added (0.2 ml per well of a 12-well plate). Adherent cells were scraped off the dish with a cell scraper and the suspension was transferred into a centrifuge tube and gently rocked for 15–30 min at 4 °C. The lysate was centrifuged at 14 000 × *g* for 20 min at 4 °C and the supernatant was immediately transferred into fresh pre-chilled micro-centrifuge tubes. The lysate was diluted at 1:4 for BCA assays with a spectrophotometer (Beckman Coulter Multimode Detector DTX880, Beckman Coulter). Finally, the lysate was aliquoted and stored at ⩽−70 °C.

### Mitochondrial oxidative phosphorylation

Mitochondrial oxidative phosphorylation profiles were determined using the human OXPHOS Magnetic Bead Panel protocol from the Milliplex Map Kit (EMD Millipore, Billerica, MA, USA). Before the assay, the samples were extracted using mitochondrial lysis buffer with protease inhibitors (EMD Millipore) and phosphatase inhibitors (EMD Millipore) according to the recommend protocol. Briefly, OXPHOS assay plates were washed with wash buffer, sealed and mixed on an orbital plate shaker for 10 min at room temperature. The wash buffer was decanted and 25 *μ*l of control, mitochondria lysis buffer and samples were added in each well. Then, 25 *μ*l beads were added into each well and incubated for 2 h at room temperature on an orbital shaker. After incubation, well contents were removed via the washing instructions provided by the protocol. Fifty microliters of detection antibodies were then added to the wells and incubated with samples for 1 h at room temperature while shaking. After incubation, well contents were removed as previously described and 50 *μ*l streptavidin-phycoerythrin was added to each well. The samples were incubated with streptavidin-phycoerythrin for 30 min at room temperature. After the incubation period, samples were washed as previously described and resuspended in Sheath Fluid. Plates were run on the Luminex MagPix machine and data were collected using the Luminex xPONENT software (v. 4.2).

### Analysis of cell proliferation

The growth curves of PDMSCs at passage 3, PDMSCs at passage 6 and DePDMSCs at passage 3 were determined using a Cell Proliferation Reagent WST-1 assay. Each group of cells was seeded at a density of 2000 cells/well in 96-well plates (Nunclon Δ Surface, Denmark). For 24, 48, 72, 96, 120, 144 or 166 h, 10 *μ*l of WST-1 reagent (5 mg/ml, Roche, Basel, Switzerland; Russia) was added to each well and cells were incubated for 1 h at 37 °C and 5% (v/v) CO_2_. Absorbance at 450 nm was measured using a spectrophotometer (Beckman Coulter Multimode Detector DTX880, Beckman Coulter). Experiments were performed in sextuplicate and repeated at least three times. In cell growth curves, the optical density (OD) values were plotted as a function of the elapsed time.

### Differentiation of PDMSCs and DePDMSCs to adipocytes, osteocytes and hepatocytes

To induce adipogenesis, PDMSCs and DePDMSCs of passage 3 at 1 × 10^5^/well in 12-well plates were treated with adipogenic medium (OriCell hMSC Adipogenic Differentiation Medium, Cyagen Biosciences) as per the manufacturer's protocol. The medium was changed three times per week. After 2 weeks, adipogenesis was confirmed by Oil red O staining (Sigma, St. Louis, MO, USA). Staining was assessed by bright-field microscopy. FABP4 and PPARG were identified in AL cells, PDMSCs and DePDMSCs at passage 1, DePDMSCs at passage 2 and DePDMSCs at passage 3 by mRNA detection. PDMSCs cultured in normal growth medium and human intraoperative abandoned adipose tissue served as controls.

For osteogenic differentiation, PDMSCs and DePDMSCs at passage 3 at 5 × 10^4^/well in 12-well plates were treated with osteogenic medium (OriCell hMSC Osteogenic Differentiation Medium, Cyagen Biosciences) as per the manufacturer's protocol. The medium was changed three times per week. After 2 weeks, osteogenic differentiation was evaluated by Alizarin Red S staining (Sigma) and detection of RUNX2 and OC mRNA. PDMSCs cultured in normal growth medium and immortalized human fetal osteoblastic cells (hFOB1.19 cell line, Cell Bank of the Chinese Academy of Sciences, Shanghai, China) served as controls.

For hepatogenic differentiation, PDMSCs and DePDMSCs of passage 3 at 1 × 10^5^/well in 12-well plates were treated with hepatogenic medium (OriCell hMSC Hepatogenic Differentiation Medium, Cyagen Biosciences) as per the manufacturer's protocol. After induction for 8 and 20 days, hepatogenic differentiation was evaluated by alpha-fetoprotein (AFP), cytokeratin 18(CK18), and cytokeratin 19 (CK19) immunocytochemical staining and detection of ALB, CYP1A2 and CYP3A4 mRNA. PDMSCs cultured in normal growth medium and human liver tissue served as controls.

### Immunocytochemistry

Cell samples were fixed in 4% (v/v) paraformaldehyde solution (MultiSciences Biotech, Hangzhou, China) at room temperature for 15 min. Endogenous peroxidase was inhibited by immersion in a 0.3% (v/v) hydrogen peroxide (H_2_O_2_) in methanol bath for 15 min. After washing, nonspecific binding was blocked with 5% (m/v) bovine serum albumin in PBS. The cells were incubated with diluted primary antibody Anti-SSEA4 antibody (15 *μ*g/ml; Abcam), Anti-TRA-1-60 (R) antibody (1 : 100; Abcam), Anti-OCT4 antibody (5 *μ*g/ml; Abcam), Anti-NANOG antibody (1 : 1000; Abcam), Anti-SOX2 antibody (1 *μ*g/ml; Abcam), Anti-FABP4(1 : 1000; Abcam), Anti-PPAR gamma (5 *μ*g/ml; Abcam), Anti-RUNX2 (1 : 500; Abcam), Anti-Osteocalcin (10 *μ*g/ml; Abcam), Anti-human AFP (1 : 200; Abcam), Anti-human CK18 (2 mg/ml, 1 : 400; Abcam), Anti-human CK19 (1 : 400; Abcam). According to the manufacturer's instructions, detection of primary antibody was performed using horseradish peroxidase-conjugated secondary antibodies (1 mg/ml, 1 : 1000; Abcam). Peroxidase activity was revealed by a 3- to 5-min exposure to diaminobenzidine tetrahydrochloride solution (DAB kit, Vector Labs, Burlingame, CA, USA). The fixed cells were then washed, counterstained with hematoxylin for 1 min, mounted and observed under an inverted phase-contrast microscope (ECLIPAS TS100, Nikon, Tokyo, Japan).

### Periodic acid-Schiff staining

The medium was taken out from culture plates and cells were washed with PBS three times. Then, the cells were fixed using 4% paraformaldehyde (MultiSciences Biotech) for 30 min. After being oxidized in periodic acid (Sigma) for 10 min and washed three times with PBS, cells were treated with Schiff's reagent (Sigma) for 15 min. Afterwards, cells were rinsed in PBS for 10 min and counterstained with hematoxylin (Sigma). The staining results were observed under an inverted phase-contrast microscope (ECLIPAS TS100, Nikon).

### Uptake and secretion of indocyanine green (ICG)

Hepatogenesis differentiation medium was replaced with L-DMEM medium containing 1 mg/ml ICG (Sigma). After incubation at 37 °C for 15 min, the cells were rinsed three times with PBS and ICG uptake was measured using an inverted microscope. Dishes were refilled with general PDMSCs medium for 6 h and color changes were examined.

### Gene expression profiling of adipogenic differentiated and dedifferentiated cells

After RNA extraction, all quantification and microarray experiments were performed at the Shanghai Biotechnology Corporation using Affymetrix PrimeView human gene expression (Affymetrix, Santa Clara, CA, USA). RNA integrity was analyzed using an Agilent Bioanalyzer 2100 (Agilent Technologies, Santa Clara, CA, USA). Qualified total RNA was further purified using an RNeasy micro kit (Qiagen, Hilden, Germany) and an RNase-Free DNase kit (QIAGEN). The RNA purity and concentration were determined using a Nanodrop 2000 (Nanodrop Products, Wilmington, DE, USA). Total RNA was amplified, labeled and purified using the GeneChip 3'IVT Express Kit (Affymetrix) following the manufacturer's instructions to obtain biotin-labeled cRNA. Array hybridization and washes were performed using the GeneChip Hybridization, Wash and Stain Kit (Affymetrix) in the Hybridization Oven 645 (Affymetrix) and Fluidics Station 450 (Affymetrix) following the manufacturer's instructions. Slides were scanned by the GeneChip Scanner 3000 (Affymetrix) and Command Console Software 4.0 (Affymetrix) with default settings. The scanned images were first assessed by visual inspection, and then analyzed to generate raw data files that were saved as CEL files using the default settings of GCOS 1.4. The raw data were normalized using an RMA algorithm in the Gene Spring Software 11.0 (Agilent Technologies). Expression profiling was performed for nine samples (*n*=3 donors) subdivided into three groups: 3 × (PDMSCs), 3 × (AL cells) and 3 × (DePDMSCs P3). The microarray data sets have been submitted to the Gene Expression Omnibus (GEO) database and are accessible via the GEO ID: GSE73964.

### Data normalization, selection criteria and analysis strategy

First, we were interested in genes whose expression was significantly up- or downregulated during the course of adipogenic differentiation. Thus, in the first step, each of the three AL cell GeneChips were compared with each of the three PDMSC GeneChips for comparative gene expression analysis. Genes were selected as differentially expressed on the basis of specific change call and fold change (FC) criteria. Changes in the *P*-value <0.01 and the FC limit >2 or <−2 were calculated for the mean FC of three comparisons to allow the selection of genes that were differentially expressed during adipogenesis. Next, we compared AL cell gene expression values with the corresponding values from DePDMSCs. The differentially expressed genes were selected according to the same criteria mentioned above. Third, we compared PDMSC gene expression values with the corresponding values from DePDMSCs. All differentially expressed genes were uploaded to the Database for Annotation, Visualization and Integrated Discovery (DAVID) 6.7 and analyzed according to the default set of statistical parameters.^53^ DAVID and the Kyoto Encyclopedia of Genes and Genomes (KEGG) were used for the evaluation and statistical analysis of genes.^54^

### Statistical analyses

The change was calculated using the comparative cycles to threshold (Ct) method. The calculation of gene expression was made as follows by comparing FABP4 expression of AL cells to PDMSCs: FABP4 (AL cells)=2^−△△Ct^; ^△△^Ct=[Ct (FABP4)−Ct (*β*-actin)] AL cells−[Ct (FABP4)−Ct (*β*-actin)] PDMSCs. All the experiments were conducted in triplicate, and the results are expressed as the mean±S.D. One-way ANOVA for three group comparison was performed by using SPSS 16.0 (SPSS, Chicago, IL, USA). *P*<0.05 was considered significant. The confidence interval was 95%.

## Figures and Tables

**Table 1a:** The enriched KEGG pathway of down- and upregulated differentially expressed genes in PDMSCs and AL cells (top five in each)

**Category**	**Description**	**Count**	**%**	***P*-value**
*Downregulated*				
KEGG	Focal adhesion	42	3.7466548	1.18E−09
KEGG	Regulation of actin cytoskeleton	41	3.6574487	3.06E−08
KEGG	Pathways in cancer	33	2.9438002	0.0648822
KEGG	MAPK signaling pathway	31	2.765388	0.0137503
KEGG	Chemokine signaling pathway	22	1.9625335	0.0360058
				
*Upregulated*				
KEGG	Pathways in cancer	26	2.8571429	0.0240712
KEGG	Lysosome	16	1.7582418	7.22E−04
KEGG	Insulin signaling pathway	13	1.4285714	0.0392537
KEGG	Jak-STAT signaling pathway	13	1.4285714	0.0914349
KEGG	Adipocytokine signaling pathway	12	1.3186813	4.57E−04

**Table 1b:** The enriched KEGG pathway of down- and upregulated differentially expressed genes in AL cells and DePDMSCs (top five in each)

**Category**	**Description**	**Count**	**%**	***P*-value**
*Downregulated*				
KEGG	Pathways in cancer	26	3.044496487	0.043737346
KEGG	Lysosome	17	1.990632319	4.34E−04
KEGG	Adipocytokine signaling pathway	12	1.405152225	7.38E−04
KEGG	TGF-beta signaling pathway	12	1.405152225	0.006228585
KEGG	Arginine and proline metabolism	9	1.053864169	0.006559557
				
*Upregulated*				
KEGG	Focal adhesion	49	3.26449034	3.56E−10
KEGG	Regulation of actin cytoskeleton	49	3.26449034	4.02E−09
KEGG	Pathways in cancer	42	2.798134577	0.028922984
KEGG	Endocytosis	27	1.798800799	0.019686088
KEGG	Tight junction	23	1.532311792	0.005755194

**Table 1c:** Three of the nine differentially expressed genes involved in multiple pathways

**Gene symbol**	**Gene name**	**Species**	**KEGG_PATHWAY**
CADM1	Cell adhesion molecule 1	*Homo sapiens*	hsa04514:Cell adhesion molecules (CAMs)
FOXO1	Forkhead box O1	*Homo sapiens*	hsa04910:Insulin signaling pathway hsa05200:Pathways in cancer hsa05215:Prostate cancer
FGF7	Fibroblast growth factor 7	*Homo sapiens*	hsa04010:MAPK signaling pathway hsa04810:Regulation of actin cytoskeleton hsa05200:Pathways in cancer hsa05218:Melanoma
WFDC1	WAP four-disulfide core domain 1	*Homo sapiens*	
MMP10	Matrix metallopeptidase 10	*Homo sapiens*	
PCDHB2	Protocadherin beta 2	*Homo sapiens*	
MAF	v-maf avian musculoaponeurotic fibrosarcoma oncogene homolog	*Homo sapiens*	
ZNF711	Zinc finger protein 711	*Homo sapiens*	
SULF2	Sulfatase 2	*Homo sapiens*	

## Abbreviations

ESCs: embryonic stem cells
iPSCs: induced pluripotent stem cells
MSCs: mesenchymal stem cells
PDMSCs: placental-derived mesenchymal stem cells
MET: mesenchymal-to-epithelial transition
BMMSCs: bone marrow mesenchymal stem cells
OXPHOS: oxidative phosphorylation
CAMs: cell adhesion molecules
ECM: extracellular matrix
DePDMSCs: dedifferentiated PDMSCs
NNT: nicotinamide nucleotide transhydrogenase
NADPH: nicotinamide adenine dinucleotide phosphate
AL: adipocyte-like
PPARG: peroxisome proliferator-activated receptor-g
ATP5A1: ATP synthase, H+ transporters, mitochondrial F1 complex, alpha subunit 1
COX4I1: cytochrome *c* oxidase subunit IV isoform 1
MT-CO1: mitochondrially encoded cytochrome *c* oxidase I
MT-CO2: mitochondrially encoded cytochrome *c* oxidase II
TFAM: transcription factor A, mitochondrial
TOMM34: translocase of outer mitochondrial membrane 34
LONP1: lon peptidase 1, mitochondrial
PPAR-a: peroxisome proliferator-activated receptor alpha
RUNX2: runt-related transcription factor 2
OC: osteocalcin
CYP: cytochrome P
ALB: albumin
BSA: bovine serum albumin
PE: phycoerythrin
FITC: fluorescein isothiocyanate
AFP: alpha-fetoprotein
CK18: cytokeratin 18
CK19: cytokeratin 19
H_2_O_2_: hydrogen peroxide
PAS: periodic acid-schiff staining
ICG: indocyanine green
FGF7: fibroblast growth factor 7
ZNF711: zinc finger protein 711
MMP10: matrix metallopeptidase 10
CADM1: cell adhesion molecule 1
