# Supplementary material for: Adipogenic placenta-derived mesenchymal stem cells are not lineage restricted by withdrawing extrinsic factors: developing a novel visual angle in stem cell biology
Source: Cell Death Dis. 2016 Mar 17;7(3):e2141–. doi: 10.1038/cddis.2016.1 (PMC4823931; doi:10.1038/cddis.2016.1)
Supplement: Supplementary Legends [file cddis20161x1.doc]

The type of Supplementary Table S1, Table S2 and were Table S3.xlsx, and the type of Supplementary Table S4 was .docx.

**Supplementary Table S1.** (**a**)We selected 2 140 out of 49 395 probe sets that represented genes with differential expression between AL cells and PDMSCs after removing double entries and probe sets with no title. (**b**) Among them, the expression levels of 952 genes were up-regulated on day 14 of adipogenesis. (**c**) Among them, the expression levels of 1 188 genes were down-regulated on day 14 of adipogenesis.

**Supplementary Table S2.** (**a**) We selected 2 486 out of 49 395 probe sets that represented genes with differential expression between AL cells and DePDMSCs. (**b**) Among them, the expression levels of 1 590 genes were up-regulated on day 21 of dedifferentiation. (**c**) Among them, the expression levels of 896 were down-regulated on day 21 of dedifferentiation.

**Supplementary Table S3.** Nine genes were differentially expressed in DePDMSCs compared to PDMSCs, all genes except for FGF7 were upregulated in DePDMSCs when compared to PDMSCs.

**Supplementary Table S4.** Primers for RT-QPCR

**Supplementary Figure S1.** Conﬁrming microarray results with RT-PCR. The expression of the PDMSC group was normalized to 1. Data are presented as the mean±S.D in triplicate and were statistically analyzed by independent t test (n = 3 independent donor cells). *=p<0.05, **=p<0.01, ***=p<0.001.
