# Supplementary figures and images for: Adipogenic placenta-derived mesenchymal stem cells are not lineage restricted by withdrawing extrinsic factors: developing a novel visual angle in stem cell biology
Source: Cell Death Dis. 2016 Mar 17;7(3):e2141–. doi: 10.1038/cddis.2016.1 (PMC4823931; doi:10.1038/cddis.2016.1)

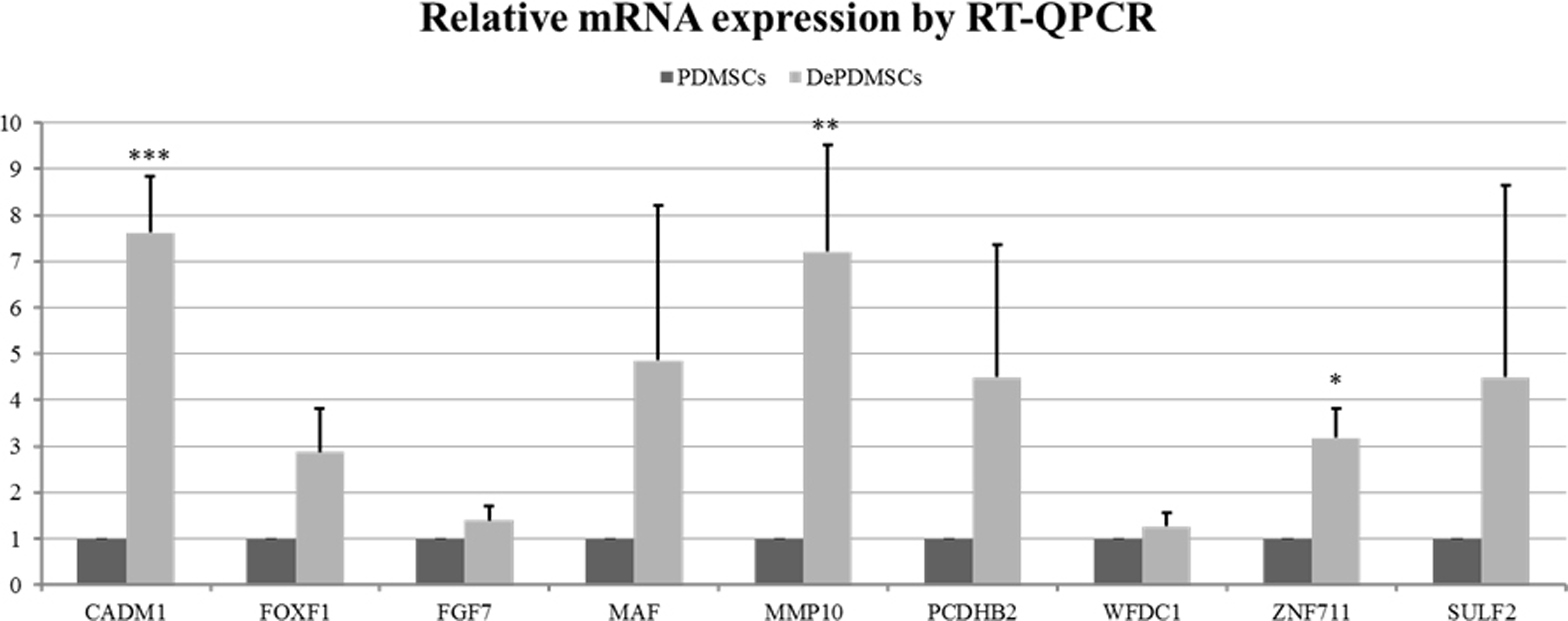

Supplement: Supplementary Figure S1 [file cddis20161x2.tif]
