# Supplementary material for: Adipogenic placenta-derived mesenchymal stem cells are not lineage restricted by withdrawing extrinsic factors: developing a novel visual angle in stem cell biology
Source: Cell Death Dis. 2016 Mar 17;7(3):e2141–. doi: 10.1038/cddis.2016.1 (PMC4823931; doi:10.1038/cddis.2016.1)
Supplement: Supplementary Table 4 [file cddis20161x6.doc]

**Supplementary Table S4**. Primers for RT-QPCR

| **Gene** | **NCBI No** | **Forward** | **Reverse** |
| --- | --- | --- | --- |
| CD29 | NM 002211.3 | 5’-TGGAGGAAATGGTGTTTGC-3’ | 5’-CGTTGCTGGCTTCACAAGT-3’ |
| CD44 | NM 000610.3 | 5’-CTGGGAGCCAAATGAAGAAA-3’ | 5’-GTGTGGTTGAAATGGTGCTG-3’ |
| CD90 | NM 006288.3 | 5’-GCACACCTCTGGCTGTCTTT-3’ | 5’-ATCCTGGCTTCCCTCTTCAC-3’ |
| CD105 | NM 000118.3 | 5’ -CCGAGTTCCTGCTCCAGTTA -3’ | 5’-CTCACACAGTTGCCCTTGG-3’ |
| FABP4 | NM 001442.2 | 5’-ACTGGGCCAGGAATTTGACG-3’ | 5’-CTCGTGGAAGTGACGCCTT-3’ |
| PPARG | NM 005037.5 | 5’-ACCAAAGTGCAATCAAAGTGGA-3’ | 5’-ATGAGGGAGTTGGAAGGCTCT-3’ |
| RUNX2 | NM 001015051.3 | 5’-TGGTTACTGTCATGGCGGGTA-3’ | 5’-TCTCAGATCGTTGAACCTTGCTA-3’ |
| OC | NM 199173.5 | 5’-GGCGCTACCTGTATCAATGG-3’ | 5’-GTGGTCAGCCAACTCGTCA-3’ |
| ATP5A1 | NM001001935.2 | 5’-CCAGGGCTATGAAGCAGGTA-3’ | 5’-AAAGTTGTTGAGTGGCAGCA-3’ |
| MT-CO1 | YP 003024028.1 | 5’-ACAGACCGCAACCTCAACAC-3’ | 5’-TCCGAAGCCTGGTAGGATAA-3’ |
| MT-CO2 | YP 003024029.1 | 5’-CATAACAGACGAGGTCAACGA-3’ | 5’-GGGCTTCAATCGGGAGTA-3’ |
| COX4I1 | NM001861.3 | 5’-GAAAGTGTTGTGAAGAGCGAAG-3’ | 5’-TGGCAGACAGGTGCTTGA-3’ |
| TFAM | NM 001270782.1 | 5’-GGTGAAATAGATAGGATGGGTTTG-3’ | 5’-GACACAGGGACTTAGGGCTTT-3’ |
| LONP1 | NM 001276479.1 | 5’-ACAGCAACGAGAACCTGGAC-3’ | 5’-CTGGCTAACGGCAATGAACT-3’ |
| TOMM34 | NM 006809.4 | 5’-CCCTTGGTGCCTGTTTCA-3’ | 5’-CTCTGGCTTTCTCCACATCC-3’ |
| PPAR-a | NM 001001928.2 | 5’-GGAGCGTTGTCTGGAGGTT-3’ | 5’-GGGAAGTGGTGGCTAAGTTG-3’ |
| ALB | NM 000477.5 | 5’-CACAGTTGCAACTCTTCGTGAAAC-3’ | 5’-AGCAGTGCACATCACATCAACC-3’ |
| CYP1A2 | NM 000761.4 | 5’-CTGGGCACTTCGACCCTTAC-3’ | 5’- TCTCATCGCTACTCTCAGGGA-3’ |
| CYP3A4 | NM 001202855.2 | 5’- AGATGCCTTTAGGTCCAATGGG-3’ | 5’- GCTGGAGATAGCAATGTTCGT-3’ |
| FGF7 | NM 002009.3 | 5’-CCTGAGCGACACACAAGAAG-3’ | 5’-GCCACTGTCCTGATTTCCAT-3’ |
| ZNF711 | NM 021998.4 | 5’-GAGACAAGGAGCCGAAGATG-3’ | 5’-TTCCCACACTCAACACAAACA-3’ |
| MMP10 | NM 002425.2 | 5’-GATGCCAGCCAAGTGTGAT-3’ | 5’-TTCAGGTTCAGGGTTCCAGT-3’ |
| CADM1 | NM 001098517.1 | 5’-GGCTTCTGCTGTTGCTCTTC-3’ | 5’-TCTGCCTGTTGGGATTCAGT-3’ |
| SULF2 | NM 001161841.1 | 5’-CCCACATCGTCCTCAACAT-3’ | 5’-TGCCTCTCTCCACCAAGAA-3’ |
| FOXO1 | NM 002015.3 | 5’-ATTCACCCAGCCCAAACTAC-3’ | 5’-GGAGGAGAGTCAGAAGTCAGC-3’ |
| WFDC1 | NM 001282466.1 | 5’-CTGTCCCTCGGGCTATGAGT-3’ | 5’-GTCGTAGGATTCGCCCATCT-3’ |
| PCDHB2 | NM 018936.3 | 5’-AGACAACGGAAGGATGGTGT-3’ | 5’-GTGACGGTGATGGTGATGTT-3’ |
| MAF | NM 001031804.2 | 5’-AAGAGGCGGACCCTGAAA-3’ | 5’-GCTGCTCACCAACTTCTCGTAT-3’ |
| β-actin | NM 001101.3 | 5’-GAGCGGGAAATCGTGCGTGACATT-3’ | 5’-GATGGAGTTGAAGGTAGTTTCGTG-3’ |
